# Epigenetic reprogramming using 5-azacytidine promotes an anti-cancer response in pancreatic adenocarcinoma cells

**DOI:** 10.1038/s41419-018-0487-z

**Published:** 2018-04-27

**Authors:** Luc Gailhouste, Lee Chuen Liew, Izuho Hatada, Hitoshi Nakagama, Takahiro Ochiya

**Affiliations:** 1 Division of Molecular and Cellular Medicine, National Cancer Center Research Institute, Tokyo, Japan; 2 Graduate School of Medicine, The University of Tokyo, Tokyo, Japan; 3 Laboratory of Genome Science, Biosignal Genome Resource Center, Institute for Molecular and Cellular Regulation, Gunma University, Maebashi, Japan; 4 National Cancer Center, Tokyo, Japan

## Abstract

Curative management of pancreatic adenocarcinoma is limited because this malignancy remains resistant to most chemotherapeutic drugs. Strategies that reverse epigenetic alterations offer a unique opportunity for cancer cell reprogramming, which is valuable for development of new treatments. The aim of this work was to reprogram pancreatic ductal adenocarcinoma (PDAC) cells toward a less aggressive and drug-responsive phenotype. The process applied is called “epigenetic reprogramming”. To evaluate the efficiency of PDAC epigenetic reprogramming, we assessed tumor growth and drug response in PANC-1 cells after exposure to non-cytotoxic doses of the demethylating agent 5-azacytidine (5-AZA). Here, we showed that an epigenetic regimen using 5-AZA promoted an anti-cancer response by inhibiting PDAC tumor growth in vivo after the engraftment of treated cells. Remarkably, the subsequent addition of gemcitabine (GEM) to the 5-AZA-mediated reprogramming resulted in a marked growth inhibition effect in GEM-resistant pancreatic cancer cells. We observed that various characteristic peptides expressed in the pancreas, which included the antiproliferative hormone somatostatin (*SST*) and the SST receptor 2 (*SSTR*2), were significantly upregulated in the epigenetically reprogrammed PDAC cells. The inhibitory effect of octreotide (OCT), an SST analog, was tested on PDAC cells and found to be improved after cell reprogramming. Furthermore, we found that *SST* gene expression restoration following 5-AZA treatment or following knockdown of the DNA methyltransferase (*DNMT*) 1 enzyme was associated with the reversion of *SST* epigenetic silencing through regional CpG demethylation. Lastly, we confirmed the efficacy of 5-AZA-based epigenetic reprogramming in vivo using a PDAC tumor growth model. In conclusion, this study demonstrates that epigenetic reprogramming using the demethylating compound 5-AZA shows anti-cancer effects in PANC-1 cells and is potentially attractive for the treatment of solid tumors.

## Introduction

Pancreatic cancer is one of the most aggressive and resistant forms of malignancy^1^. Mainly represented by pancreatic ductal adenocarcinoma (PDAC), it represents the fifth leading cause of cancer-related death in industrialized countries^2^. Diagnosis is frequently late because of the absence of disease-specific symptoms and new patients usually present with advanced or metastatic diseases. The deoxycytidine analog gemcitabine (GEM) and GEM-based combination therapies have been considered as standard treatments for limiting pancreatic cancer progression^3,4^. However, tumor ablation remains the only potentially curative option for pancreatic cancer. Given that only 15–20% of PDAC patients are considered to be appropriate candidates for surgical resection and rapidly develop local recurrence^5^, new therapeutic alternatives are urgently required.

Epigenetic regulations are crucial for orchestrating key biological events in eukaryotic organisms including embryonic development, cell differentiation, and modulation of tissue-specific gene expression^6^. Epigenetic marks, such as DNA cytosine methylation and histone modifications, help to ensure the integrity of the genome and maintain methylation states over the course of repeated cell divisions^7,8^. The significance of DNA methylation has been extensively described in cancer cells, in which oncogenes and tumor-suppressor genes acquire cancer-specific methylation patterns^9,10^. Unlike oncogenic mutations, which are permanent changes in the cancer genome, epigenetic alterations are potentially reversible, offering a unique therapeutic opportunity^11^. The cytidine analogs 5-azacytidine (5-AZA, azacytidine) and its deoxy derivative 5-aza-2′-deoxycytidine (5-AZA-dC, decitabine) have shown efficacy for the treatment of myelodysplastic syndromes^12^. Regarding the treatment of solid tumors, development of epigenetic therapies has started to regain attention despite the variable efficacies reported so far^13,14^.

The development of relevant strategies erasing “cancer imprinting” and aberrantly hypermethylated marks represents a valuable asset for the therapeutic management of pancreatic adenocarcinoma. The aim of this work was to investigate the feasibility of reversing the malignant phenotype of pancreatic cancer cells by epigenetic reprogramming using the human PDAC cell line PANC-1. We first evaluated PANC-1 cell growth in response to 5-AZA treatment in vitro to determinate the optimal concentration for cell reprogramming. Next, PDAC tumor growth was analyzed in vivo after the engraftment of epigenetically reprogrammed PANC-1 cells into mice to validate the efficiency of the procedure. Importantly, we investigated whether 5-AZA-based epigenetic reprogramming could potentiate the cytotoxic effect of the chemotherapeutic agent GEM on resistant PDAC cells. In addition, we explored the molecular mechanism underlying the reversion of the epigenetic silencing of characteristic markers expressed the pancreas, in particular for the antiproliferative hormone somatostatin (*SST*), which was seen in reprogrammed pancreatic cancer cells. To this end, the correlations between the expression and methylation profiles of the *SST* gene were analyzed after 5-AZA-mediated epigenetic reprogramming and DNA methyltransferase (*DNMT*) 1 knockdown. Lastly, we assessed the potential anti-cancer action of an epigenetic regimen on PDAC tumors in vivo.

## Results

### Exposure to the epigenetic drug 5-AZA inhibits PDAC tumor growth

**Fig. 1:**
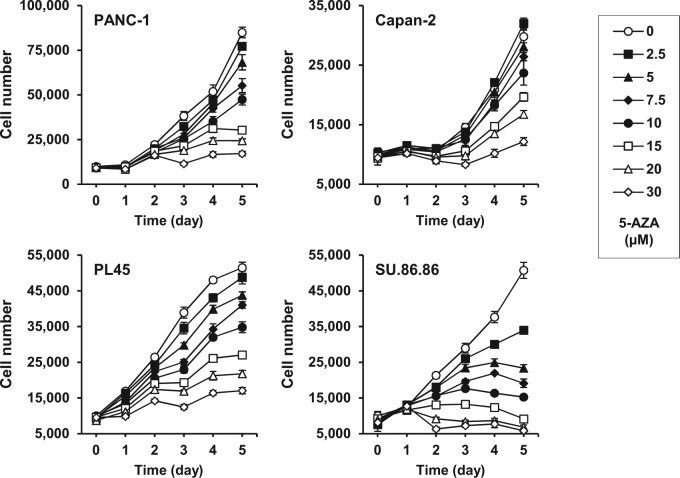
Inhibition of PDAC cell growth in response to 5-AZA treatment. Time and dose-dependent cytotoxicity of 5-AZA as evaluated in the four human PDAC cell lines PANC-1, Capan-2, PL45, and SU.86.86. Twenty-four hours after seeding, cells were treated with 5-AZA at the indicated concentrations (day-0). 5-AZA medium was prepared and replaced daily. Cell number was estimated at the indicated times using a cell viability assay (MTT). The data depicted show the mean ± standard deviation (SD) and are representative of three distinct experiments

To investigate the prospective therapeutic use of epigenetic reprogramming in pancreatic adenocarcinoma, we first evaluated the effect of the demethylating agent 5-AZA on the four human PDAC cell lines PANC-1, Capan-2, PL45, and SU.86-86. Cell viability assays were performed and showed a clear dose–response effect, resulting in a gradual decrease in cell growth and significant toxicity after 3 days for the PDAC cells that had been treated with high doses of 5-AZA (Fig. 1). Calculation of 5-AZA IC50 showed that Capan-2 and PANC-1 cells were the most resistant to the demethylating drug, with an IC50 of 71.3 and 45.6 µM, respectively, after 48 h of exposure. Conversely, SU.86.86 cells appeared more responsive to 5-AZA (IC50 = 19.2 µM, 48 h).

**Fig. 2:**
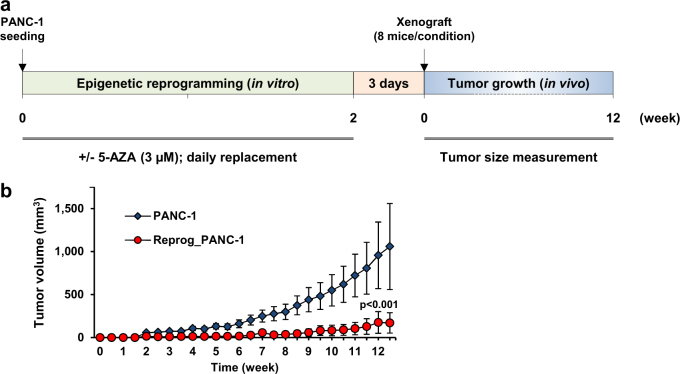
Suppression of PANC-1 cell tumorigenicity after exposure to non-cytotoxic doses of 5-AZA. **a **Experimental design for the assessment of in vivo PDAC cell growth after epigenetic reprogramming. PANC-1 cells were pretreated with 3 µM 5-AZA for 2 weeks (in vitro). 5-AZA medium was prepared and replaced daily. After 3 days without 5-AZA, the reprogrammed and control cells were subcutaneously implanted in athymic nude mice. An equal number of viable cells was injected for each inoculum. The tumor nodules were monitored twice a week for 12 weeks (in vivo). **b** Xenograft tumorigenicity assay following the epigenetic reprogramming of PANC-1 cells. The data depicted show the mean ± standard error of the mean (SEM). The *p* value was calculated with a *t*-test to statistically evaluate the difference in tumor growth between the control (*N* = 8) and the reprogrammed PANC-1 cell group (*N* = 8)

Next, we assessed the in vivo tumorigenic ability of PDAC cells after epigenetic reprogramming. Experiments were carried out using the PANC-1 cell line because these cells exhibited one of the most aggressive phenotypes among the four PDAC cell lines previously characterized. Based on our experience, the PANC-1 cells also showed tumorigenic ability in vivo. The concentration of 5-AZA used for the epigenetic reprogramming of PANC-1 cells was determined based on the MTT assays and IC50 values to minimize the cytotoxic effect of the compound. Accordingly, PANC-1 cells were implanted into mice after a 2-week reprogramming regimen using 3 µM 5-AZA with daily replacement, and tumor size was monitored for 12 weeks (Fig. 2a). As a result, a significant and persistent inhibition of tumor growth was observed with the epigenetically reprogrammed cells compared with the PANC-1 cells that were not treated prior to inoculation (*p* < 0.001, *t*-test) (Fig. 2b). Remarkably, the reprogrammed PANC-1 cells nearly lost their ability to form tumors in vivo as tumor nodules were barely measurable up to 9 weeks after cell engraftment.

### Epigenetic reprogramming with non-cytotoxic doses of 5-AZA sensitizes PANC-1 cells to gemcitabine

**Fig. 3:**
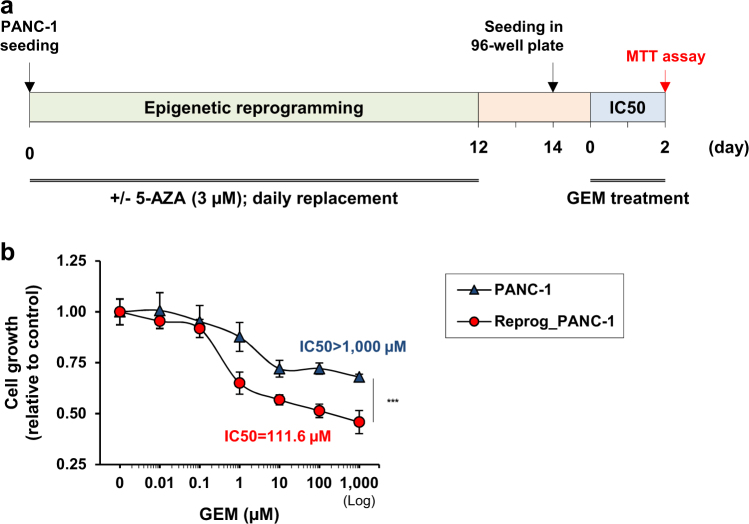
Increased response to GEM treatment in epigenetically reprogrammed PANC-1 cells. **a** Experimental design for the measurement of GEM cytotoxicity on PANC-1 cells after epigenetic reprogramming. The PANC-1 cells were pretreated with 3 µM 5-AZA for 2 weeks with daily replacement. After 48 h without 5-AZA, the reprogrammed and control cells were seeded in 96-well plates. Twenty-four hours after seeding, GEM was added to the medium without 5-AZA and cell viability was measured using an MTT assay. **b** IC50 of GEM in the reprogrammed and control PANC-1 cells. Cell viability was measured at the indicated concentrations 48 h after the beginning of GEM treatment to determine the IC50 of the compound. Control PANC-1 cells showed low response to GEM (IC50 > 1000 µM). GEM IC50 was significantly reduced in epigenetically reprogrammed PANC-1 cells compared with the control cells (IC50 = 111.6 µM; *p* < 0.001 using the *t*-test). The statistical significance of the differences between the reprogrammed and control PANC-1 cells was ***p < 0.001 (*t*-test). All data shown in the figure are mean ± SD

We evaluated whether 5-AZA-based epigenetic reprogramming could potentiate the cytotoxic effect of the chemotherapeutic agent GEM in resistant pancreatic cancer cells. First, PANC-1 cells were reprogrammed by 5-AZA treatment (3 µM) for 2 weeks (Fig. 3a). Following this epigenetic reprogramming regimen, the cells were reseeded without 5-AZA and treated with increasing concentrations of GEM. Importantly, we confirmed that a 2-week reprogramming of PANC-1 cells using 3 µM 5-AZA did not significantly affect cell viability (Supplementary Figure 1). The growth inhibitory effect of GEM on PANC-1 cells was assessed by an MTT assay after 48 h of treatment, and the IC50 was calculated. Measurement of cell growth confirmed that non-reprogrammed PANC-1 cells were resistant to GEM with an IC50 greater than 1000 µM (Fig. 3b). Conversely, growth of epigenetically reprogrammed PDAC cells was significantly inhibited by GEM in a concentration-dependent manner, with an IC50 equivalent to 111.6 µM after 48 h of treatment (*p* < 0.001 compared with the control cells, *t*-test). While GEM had a limited effect on PANC-1 cells, the sequential combination of 5-AZA-based epigenetic reprogramming and GEM increased sensitivity of PDAC cells toward GEM in vitro.

### 5-AZA-based epigenetic reprogramming enhances *SST* expression in PANC-1 cells and restores SST analog response

**Fig. 4:**
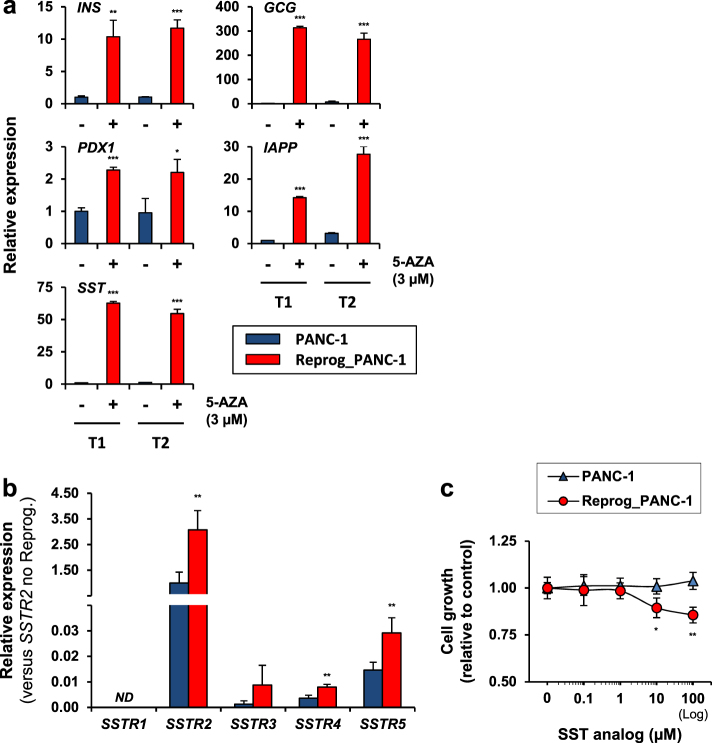
Effect of 5-AZA-mediated epigenetic reprogramming on *SST* and *SSTR* gene expression, and SST analog response. **a** Relative expression levels of four major endocrine lineage markers, insulin (*INS*), glucagon (*GCG*), amylin (islet amyloid polypeptide, *IAPP*), and somatostatin (*SST*), and the insulin promoter factor (pancreatic and duodenal homeobox 1, *PDX1*) in epigenetically reprogrammed PANC-1 cells. The total RNAs were extracted from the PANC-1 cells after reprogramming with 3 µM 5-AZA for 14 and 16 days (T1 and T2, respectively), and the relative mRNA expression levels were determined by RT-qPCR. The non-reprogrammed PANC-1 cells were used as controls. Statistically significant differences in the gene expression levels were achieved at *p < 0.05, **p < 0.01, and ***p < 0.001 (*t*-test). **b** Expression of somatostatin receptors (*SSTR*s) in reprogrammed and control PANC-1 cells. Relative mRNA levels are expressed with regard to the expression level of SSTR2 measured in the non-reprogrammed PANC-1. Statistical significance: **p < 0.01 (*t*-test). ND not detected. **c** Evaluation of SST analog effect on reprogrammed PDAC cell growth. Cell viability was measured 120 h after starting treatment with the SST analog OCT at the indicated concentrations. Statistical significance: *p < 0.05 and **p < 0.01 (*t*-test). All data shown in the figure are mean ± SD

To assess the molecular phenotype of PANC-1 cells in response to the 5-AZA-mediated epigenetic reprogramming, the expression level of several endocrine markers was analyzed by RT-qPCR. Significant differences were obtained with some of the most characteristic peptides produced by the pancreas, such as insulin (*INS*), glucagon (*GCG*), amylin (islet amyloid polypeptide, *IAPP*), and *SST*, which were all consistently upregulated in response to 5-AZA treatment (Fig. 4a). In this study, *SST* was considered for further investigation because of the potential tumor-suppressor activity of this antiproliferative hormone^15,16^. We observed that the mRNAs of *SST* were at extremely low levels in non-reprogrammed PDAC cells, whereas *SST* expression was remarkably increased in 5-AZA-treated cells, with an induction ratio greater than 55-fold (*p* < 0.001, *t*-test). Using the publicly available data sets from the Human Protein Atlas Program, we confirmed that SST protein is expressed in normal pancreatic tissues, but strongly repressed in pancreatic tumors (Supplementary Figure 2). Next, we quantified the mRNA levels of the five human somatostatin receptors (*SSTRs*) before and after epigenetic reprogramming. As shown in Fig. 4b, *SSTR2*, *SSTR3*, *SSTR4*, and *SSTR5* but not *SSTR1* were expressed in PANC-1 cells. Among the four detected receptors, *SSTR2* exhibited the highest expression, whereas *SSTR3*, *SSTR4*, and *SSTR5* were expressed at similar low levels. The epigenetically reprogrammed cells showed a significant 3.1-, 2.2-, and 2.0-fold induction of *SSTR2*, *SSTR4*, and *SSTR5* mRNA, respectively, compared with the control cells (*p* < 0.001, *t*-test).

SST analogs have been used for treating gastroenteropancreatic neuroendocrine tumors, a rare form of malignancy^17^. However, assessment of the therapeutic index of SST analogs in the management of PDAC tumors are still needed^18^. Here, we evaluated the effect of the SST analog octreotide (OCT) on PDAC cell growth. As previously described, PANC-1 cells were reprogrammed by a 5-AZA regimen (3 µM for 2 weeks) before the beginning of the treatment with different dosages of OCT. We observed that PANC-1 cells were resistant to OCT and did not show any modification of their growth. Conversely, epigenetic reprogramming was able to improve OCT-induced PANC-1 cell response (Fig. 4c). Although the growth inhibitory effect of the SST analog observed in the reprogrammed PDAC cells was moderate, statistical significance was reached, with an inhibition of 10.5 ± 5.2 and 14.4 ± 4.2% using 10 and 100 µM OCT, respectively (*p* < 0.05 and *p* < 0.01 compared with the control reprogrammed cells not exposed to OCT, *t*-test).

### 5-AZA treatment and *DNMT1* knockdown reverts the epigenetic silencing of *SST*

**Fig. 5:**
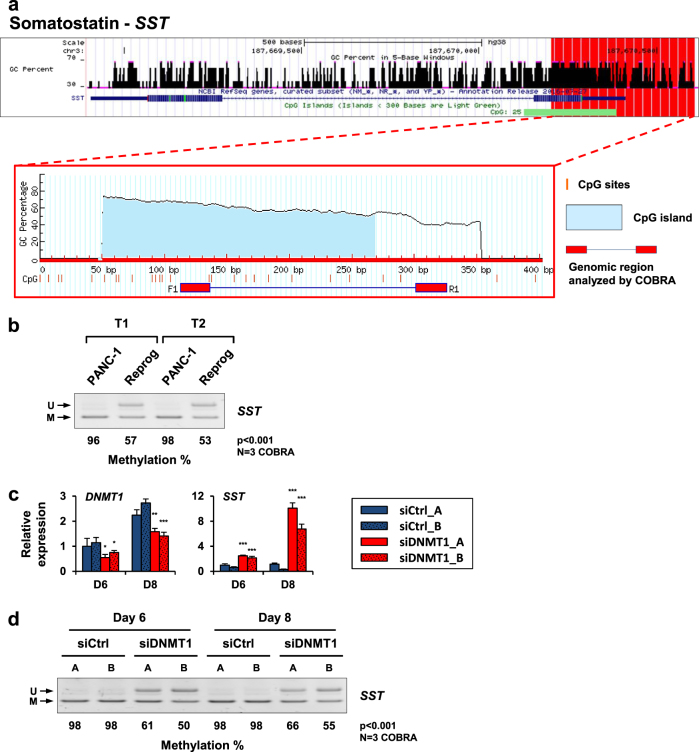
Expression levels and methylation profiles of *SST* after 5-AZA treatment and *DNMT1*-knockdown. **a** In silico analysis of the human *SST* gene. The figures show the GC percentages, CpG sites, and the COBRA-amplified genomic region. The CpG sites in *SST* promoter were identified using the UCSC Genome Bioinformatics tool. The *SST* gene exhibits a CpG island of 262 bp (green horizontal bar), which contains 25 CpG sites. **b** COBRA was performed to evaluate CpG methylation (%) in the promoter of the *SST* gene in the control and epigenetically reprogrammed PANC-1 cells. Reprogrammed cells were treated with 5-AZA (3 µM) for 14 and 16 days (T1 and T2, respectively) before genomic DNA extraction. Representative data of three COBRA are shown. **c** Relative expression of *DNMT1* and *SST* following *DNMT1* silencing in PANC-1 cells. Two distinct siRNAs were used to target *DNMT1* (siDNMT1_A and B), and two scrambled siRNAs were used as negative controls (siCtrl_A and B). The histograms show the mean ± SD of *SST* and *DNMT1* expression levels, measured 6 and 8 days after transfection. **d** *SST* promoter methylation percentage after *DNMT1* knockdown, as determined by COBRA. The data are representative of three COBRA. Genomic DNA was extracted from PANC-1 cells 6 and 8 days after transfection. Statistical significance: *p < 0.05, **p < 0.01, and ***p < 0.001 (*t*-test). U unmethylated, M methylated

To address whether 5-AZA acts by directly influencing the methylation state of the *SST* gene, the correlation between DNA methylation and *SST* expression levels was evaluated in reprogrammed and control PDAC cells. In silico genomic analysis revealed that *SST* contained a CpG-rich region in its promoter (Fig. 5a). We performed combined bisulfite restriction analysis (COBRA) to examine the methylation status of the identified CpG sites and found prominent hypermethylation of the *SST* promoter in PANC-1 cells, with a methylation rate of nearly 100% (Fig. 5b). By contrast, reprogrammed PANC-1 cells exhibited significant demethylation of the analyzed CpG sites (*p* < 0.001, *t*-test), which was consistent with the reexpression of the *SST* gene observed after 5-AZA treatment. Interestingly, the COBRA data also identified a correlation between the restoration of *INS* expression in 5-AZA-treated cells (Fig. 4a) and demethylation of the CpG sites located in *INS* promoter (Supplementary Figure 3a and 3b).

CpG methylation is primarily controlled by three major DNMT enzymes, of which DNMT1 plays a critical role in the maintenance of methylation patterns during cellular replication^8,19^. The expression and methylation profile of *SST* was evaluated after siRNA-based silencing to substantiate the impact of *DNMT1* repression in the epigenetic reprogramming process. As a result, the specific knockdown of *DNMT1* was correlated with a significant increase in *SST* expression levels (Fig. 5c). In addition, the COBRA data revealed that *SST* reexpression was accompanied by a substantial demethylation of the CpG sites located in its promoter region (Fig. 5d). A similar result was observed regarding the expression and methylation level of the *INS* gene after *DNMT1* experimental silencing (Supplementary Figure 3c and 3d), demonstrating the efficiency of the *DNMT1* siRNA in mimicking the effects of 5-AZA treatment. Taken together, these data implicated *DNMT1* in the maintenance of *SST* epigenetic silencing in pancreatic adenocarcinoma and supported the contribution of 5-AZA-mediated *DNMT1* inactivation, which could be responsible for the demethylation of the *SST* promoter and *SST* reexpression in epigenetically reprogramed PANC-1 cells.

### Epigenetic reprogramming regimen shows efficacy in vivo and suppresses PANC-1 tumor growth

**Fig. 6:**
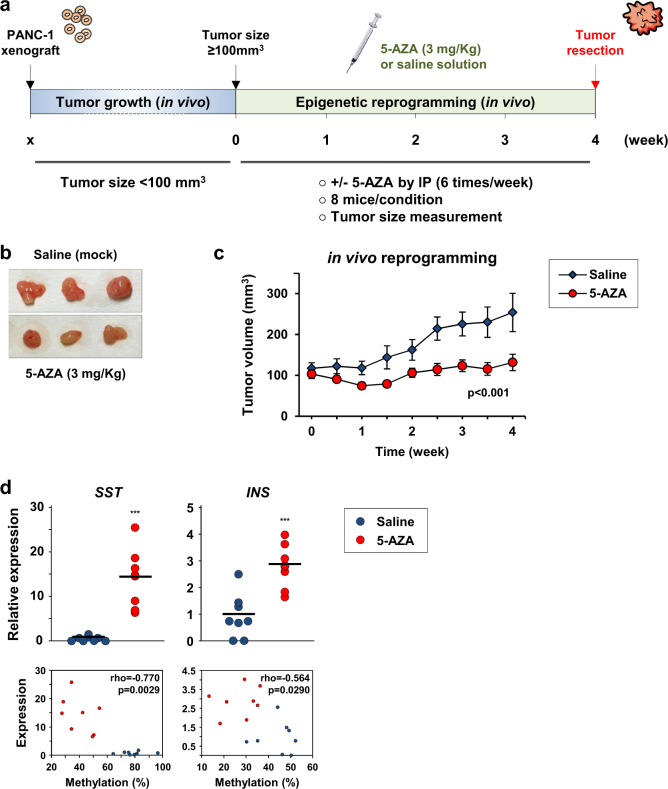
Efficacy of epigenetic reprogramming regimen in repressing PANC-1 tumor progression in vivo. **a** Schematic outline that illustrates the epigenetic reprogramming procedure. The PANC-1 cells were used to generate tumors in nude mice. In vivo cell reprogramming was performed by treating the mice with 5-AZA for 4 weeks (6 times/week) at a dose of 3 mg/kg by intraperitoneal injection (IP). Saline solution was injected for the control group. See Supplementary Figure 4 for survival curves and animal weight in response to 5-AZA exposure. **b** Representative size of the tumors at the end of the experimental protocol. Saline-treated mice: 253.9 ± 124.0 mm^3^. 5-AZA-treated mice: 131.7 ± 75.2 mm^3^. **c** PDAC tumor growth in response to 5-AZA treatment in vivo. The size of the tumor nodules in the reprogrammed (*N* = 8) and control group (*N* = 8) were monitored twice a week for 4 weeks. The data show the mean ± SEM. Statistical significance was evaluated with the *t*-test. **d** Relative expression levels and methylation profiles of *SST* and *INS* after epigenetic reprogramming. The total RNAs and genomic DNAs were extracted for analysis from the resected tumors (4 weeks of treatment). The horizontal bars show the averages and statistical significance was ***p < 0.001 (*t*-test). The **s**catter plots show the correlation between the expression of *SST* and *INS* and CpG methylation levels of their respective promoters (Spearman’s rank coefficient). The red and blue dots show the 5-AZA-treated mice (*N* = 8) and saline-treated mice (*N* = 8), respectively

To evaluate the relevance and consistency of the epigenetic reprogramming regimen, we tested whether 5-AZA treatment could modify the malignant phenotype of tumors generated from PDAC cells in vivo. A pilot dose–response assay with daily injections determined that 5-AZA concentrations up to 3 mg/kg did not significantly affect animal survival (Supplementary Figure 4a) and body weight (Supplementary Figure 4b). PANC-1 cells were used and implanted into nude mice as xenograft models. 5-AZA treatment started when tumors reached a palpable size (≥100 mm^3^). The epigenetic therapeutic procedure was based on an intraperitoneal (IP) injection of 3 mg/kg 5-AZA, 6 times/week for 4 weeks, at which point the mice were euthanized and the tumors collected (Fig. 6a). At the end of treatment, the tumors appeared to be markedly smaller in response to injections of the demethylating agent (Fig. 6b). Thus, the average size of the resected tumors was 253.9 ± 124.0 and 131.7 ± 75.2 mm^3^ for saline- and 5-AZA-treated mice, respectively. As presented in Fig. 6c, nodule size monitoring showed a significant inhibition of tumor progression from 2.5 weeks of treatment (*p* < 0.001, *t*-test, *N* = 8 mice per group).

To confirm that 5-AZA-mediated epigenetic reprogramming was effective in vivo, *SST* and *INS* expression levels were analyzed by RT-qPCR in the PDAC tumor tissues after treatment. After measurement, *SST* and *INS* mRNA levels were found to be relatively low in the untreated mice, which confirmed the silencing of these genes in the tumors generated from the PANC-1 cells (Fig. 6d). Conversely, 5-AZA treatment was associated with a remarkable reexpression of these two endocrine peptides (*p* < 0.001, *t*-test). The subsequent observations demonstrated that the increased expression of *SST* and *INS* was consistent with the demethylation of their respective promoters, as shown by the COBRA data. Spearman’s rank correlation analyses revealed that the CpG methylation ratios were inversely correlated with the expression levels of *SST* (*ρ* = −0.770, *p* = 0.0029) and *INS* (*ρ* = -0.564, *p* = 0.0290) in PDAC tumors. Consequently, these results indicated that the correlation between the reversion of epigenetic silencing and 5-AZA treatment was effective in vivo.

## Discussion

So far, progress in treating pancreatic cancer has been limited because of the poor response of PDAC cells to chemotherapies. In the present work, we reported that an epigenetic therapy regimen using the demethylating agent 5-AZA significantly inhibited pancreatic tumor growth and sensitized the PDAC cell line PANC-1 to GEM and SST analog treatment. Furthermore, we showed that epigenetic silencing of the antiproliferative hormone *SST* was reverted in epigenetically reprogrammed PDAC cells.

In humans, ~70% of annotated gene promoters contain CpG-rich regions, which might be potentially affected by “cancer imprinting”^20^. Our current knowledge indicates that abnormal DNA methylation is usually observed in pancreatic neoplasms and is correlated with the progression of the disease^21–23^. However, the epigenetic silencing of *SST* and its reversion by epigenetic reprogramming has never been described in pancreatic cancer cells. In addition, this work is the first to report the therapeutic potential of a demethylating compound using an in vivo model of pancreatic ductal adenocarcinoma.

SST acts as an endogenous inhibitory regulator of various cellular functions including hormone secretion, cell motility, and cell proliferation^15,16^. Although SST analogs have been used for treating particular pancreatic neuroendocrine malignancies^17^, their efficacy for PDAC tumor management would require further investigations and considering *SSTR* status^18^. Consistent observations regarding the epigenetic silencing of the antiproliferative peptide SST have been seen in various types of malignancy. For example, *SST* promoter hypermethylation was reported as a common event in human esophageal carcinoma and was related to early neoplastic progression in Barrett’s esophagus^24^. Several studies have demonstrated the decrease in cell proliferation induced by SST and SST analogs in SSTR2-positive pancreatic cancer cells^16,25^. Using the PANC-1 cell line, the group of Li and colleagues revealed a synergistic inhibitory effect on cell growth mediated by SST analog treatment after SSTR1 and SSTR2 reexpression^26^. In our study, epigenetic reprogramming of PANC-1 cells was associated with an increase in *SSTR2* expression and a significant inhibition of cell growth in response to the SST analog OCT. Previous data indicated the frequent downregulation of *SSTR*s in pancreatic adenocarcinoma tissues and derived cell lines^27,28^. Torrisani and coworkers found an upstream promoter of *SSTR2* that was controlled by CpG methylation and was hypermethylated in various pancreatic cell lines, including PANC-1 cells^29^. *SSTR1* gene inactivation through a similar epigenetic mechanism was also identified in Epstein-Barr virus-positive gastric cancer^30^. We performed in silico analyses and confirmed the presence of large CpG islands in the coding sequences of the five *SSTR* genes (Supplementary Figure 5).

Surprisingly, we found that other endocrine lineage genes were significantly increased in 5-AZA-treated PDAC cells, which included *INS*, *GCG*, and *IAPP*. As evidenced for *SST*, the reexpression of *INS* was the consequence of demethylation of the CpG sites located in the promoter of this gene. In line with this finding, Lefebvre and colleagues previously showed that 5-AZA-dC was able to induce Ngn3, a major marker for islet progenitors, and endocrine differentiation in PANC-1 cells^31^. However, we were not able to detect INS protein in reprogrammed PANC-1 cells using the ELISA method (data not shown). It is reasonable to hypothesize that SST augmentation observed in our cells might participate in the alteration of INS production at the protein level because of its inhibitory effect. Nevertheless, our data are encouraging and support the importance of further developing epigenetic methods to induce the differentiation of PDAC cell lines toward an endocrine-like lineage in order to generate relevant insulin-producing human cell models.

In our study, the specific depletion of the DNMT1 enzyme was able to mimic 5-AZA-mediated reprogramming and increased expression of *SST* and *INS*. *DNMT1* expression is known to be frequently increased in PDAC tumors and associated with poor prognosis. For example, Wang and coworkers examined the expression of *DNMT1* by immunohistochemistry staining in PDAC and benign pancreatic tissues and found that DNMT1 protein levels increased from precursor to advanced lesions^32^. Another study evaluated *DNMT1* gene expression in 88 PDAC tumors and 10 normal pancreatic tissues and showed that *DNMT1* was expressed in 46.6% of PDAC tissues but not in the normal pancreatic tissues analyzed^33^. We previously reported the inverse correlation between *DNMT1* expression and the tumor-suppressor microRNA-148a in liver cancer^34^. Similarly, Robert and collaborators demonstrated that *DNMT1* was required to maintain aberrant CpG methylation in colon cancer cells^35^. The authors showed that *DNMT1* knockdown significantly promoted the ability of 5-AZA-dC to reactivate the tumor-suppressor genes silenced by hypermethylation.

Therapeutic strategies using DNA demethylating agents represent attractive alternatives for the treatment of solid tumors^36^. Here, we show that 5-AZA exerted its antitumor effect by reducing the tumorigenic potential of PANC-1 cells while also increasing GEM treatment response. Importantly, our data demonstrated that a pretreatment model rather than the use of a combination of two drugs at once was able to reprogram PDAC cells to leave them primed for killing by another anti-cancer agent, such as GEM. Recent data support the idea that reactivation of specific genes by hypomethylation drugs holds the key to therapeutic benefits in non-small cell lung cancer^37^, melanoma^38^, glioma^39^, hepatoma^40^, and epithelial tumors^41^. Previous studies in pancreatic cancer have also reported the use of the deoxy derivative of 5-AZA, 5-AZA-dC. However, the effect of 5-AZA itself on PDAC tumor growth and GEM-drug resistance has been poorly investigated. Missiaglia and colleagues showed that 5-AZA-dC treatment resulted in global DNA demethylation and apoptosis of pancreatic cancer cell lines^42^. More recently, 5-AZA-dC was tested in an aggressive stroma-rich mouse model of pancreatic adenocarcinoma^43^. Wang and coworkers also showed that the MEK inhibitor PD98059 potentiated the capability of 5-AZA-dC to mediate growth arrest in pancreatic cancer cells^44^. Currently, a phase II trial is recruiting participants to determine the effect of 5-AZA (oral azacytidine) on progression-free survival and outcomes in patients with resected pancreatic adenocarcinoma at high risk of recurrence^45^.

The establishment of epigenetic therapies for solid tumor treatment will remain challenging. First, given that our study was performed using one PDAC cell line, additional experiments using other cancer cell types and other DNA demethylating drugs will be required to evaluate the potential clinical application of epigenetic reprogramming-based therapies. Next, it will be critical to accurately determine the optimal dosage of the demethylating agents to maximize the possibility of long-term treatment and ensure patient response and tolerance. In addition, further investigations will be required to address the specificity of cancer cell reprogramming with regard to undesirable gene reexpression and possible side effects on non-neoplastic cells. Even though epigenetic drugs seem to preferentially reactivate genes that have been abnormally silenced in cancer cells^46^, the reason why these cancer imprinted genes are more susceptible to reactivation by demethylation treatment remains unclear.

In summary, our study emphasizes an effective method for the epigenetic-based reprogramming of pancreatic adenocarcinoma cells. We demonstrate that epigenetically reprogrammed PANC-1 cells using 5-AZA exhibit a less aggressive phenotype with impaired tumor growth and improved GEM response. The results from ongoing investigations will be essential to determine the therapeutic value of epigenetic compounds for potential applications in the treatment of solid tumors. Nevertheless, it is appealing to consider that such a reprogramming strategy may pave the way for further controlling aggressive cancers and promote development of alternative therapies for inoperable or drug-resistant tumors.

## Materials and methods

### Cells and reagents

The human pancreatic adenocarcinoma cell lines, Capan-2, PL45, and SU.86.86, were purchased from the American Type Culture Collection. The PANC-1 cells were obtained from the Public Health England Culture Collection. Cultured PANC-1 and PL45 cells were maintained in DMEM (Gibco) supplemented with penicillin (50 IU/mL; Gibco), streptomycin (50 µg/mL; Gibco), and 10% fetal bovine serum (FBS; Thermo Scientific). Capan-2 and SU.86.86 cells were cultured in McCoy’s 5 A (Gibco) and RPMI 1640 medium (Gibco), respectively, and both were supplemented with penicillin (50 IU/mL; Gibco), streptomycin (50 µg/mL; Gibco), and 10% FBS. The demethylating agent 5-azacytidine (5-AZA; PubChem CID: 9444) was from Sigma (#A2385). The drug was dissolved in phosphate-buffered saline as a 10 mM stock, filtered (0.22 µM), and stored at −20 °C in aliquots that were thawed immediately prior to use. The in vitro epigenetic reprogramming procedure was performed by addition of 5-AZA to the PANC-1 cells at a concentration of 3 µM. Given the short half-life of the compound in culture media, 5-AZA medium was prepared and replaced daily. Gemcitabine (GEM; PubChem CID: 60750) and the SST analog octreotide (OCT; PubChem CID: 448601) were purchased from Sigma (#G6423 and O1014, respectively). The compounds were dissolved in H_2_O as a 10 mM stock solution for GEM and 1 mM stock solution for the SST analog, filtered (0.22 µM), and stored at −20 °C.

### Cell growth assay and IC50

For the evaluation of the time and dose-dependent cytotoxicity of 5-AZA, PANC-1 cells were seeded at 7,500 cells per well in 96-well plates and Capan-2, PL45, and SU.86.86 cells were seeded at 10,000 cells/well (6 wells/condition). The next day, the medium was changed and cells were treated with the indicated concentrations of 5-AZA for one to five days (daily replacement). Cell viability was measured at the indicated times using the Cell Counting Kit-8 (Dojindo), according to the manufacturer’s instructions (MTT assay). The absorbance at 450 nm was measured using the Synergy H4 Microplate Reader system (BioTek). For the evaluation of concentration-dependent cytotoxicity of GEM (IC50), reprogrammed and control PANC-1 cells were seeded in 96-well plates (10,000 cells/well; 6 wells/condition). The next day, the medium was changed, and reprogrammed and control PANC-1 cells were cultured in medium containing different concentrations of GEM for 48 h. Treatment with 5-AZA was discontinued two days before seeding in 96-well plates, and cells were maintained without 5-AZA until the end of the experiments. Cell viability was measured as mentioned above. A similar protocol was used for the assessment of the effect of the SST analog on PANC-1 cell growth before and after epigenetic reprogramming.

### Xenograft establishment and tumorigenicity assay

Female athymic nude mice were purchased at 4–5 weeks old and housed in isolator units under controlled humidity and temperature, with a 12-h light–dark cycle. The animals received standard sterilized food and water ad libitum. The epigenetically reprogrammed cells (in vitro reprogramming) and control PANC-1 cells were subcutaneously implanted into the right flanks of the mice at a density of 8 × 10^6^ cells by inoculation in DMEM without serum (100 µL/mouse). The tumor nodules were monitored twice a week by palpation using a digital caliper. The tumor size was determined using the formula (length × width²)/2 (mm^3^). The experiments continued until tumors reached the maximum allowable size dictated by the animal care guidelines of our institute. Animal experimental protocols were approved by the National Cancer Center Institutional Animal Care and Use Committee.

### Cell transfection

The PANC-1 cells were seeded at a density of 40,000 cells/cm² in 35-mm-diameter culture dishes and transfected the next day using the TransFectin lipid reagent (Bio-Rad Laboratories). The cells were incubated with the transfection mix containing 100 nM of siRNA and 5 µL of TransFectin in a 1.2 mL total volume of serum- and antibiotic-free OptiMEM (Invitrogen) for 5 h. The two human *DNMT1* siRNAs were purchased from Ambion (ID #s4215 and #s4217; siDNMT1_A and B, respectively). The two control siRNAs, AllStars Negative Control (ID #1027281; siCtrl_A) and Silencer Select Negative Control siRNA (ID #4390843; siCtrl_B), were purchased from Qiagen and Life Technologies, respectively.

### Total RNA and genomic DNA isolation

The mRNAs were purified using the miRNeasy Mini Kit (Qiagen), according to the manufacturer’s protocol. The total RNAs were quantified using a NanoDrop 1000 spectrophotometer (Thermo Scientific), and the integrity of the RNA was evaluated with an Agilent 2100 Bioanalyzer (Agilent Technologies). The genomic DNA was extracted using the GenElute Mammalian Genomic DNA Miniprep Kit (Sigma) and was quantified on a NanoDrop 1000 spectrophotometer.

### Real-time reverse transcription quantitative polymerase chain reaction (RT-qPCR)

To evaluate the gene expression levels, the total RNAs were first treated with DNase using the TURBO DNA-free kit (Ambion). Then, cDNAs were synthesized from 1 µg of purified mRNA using SuperScript III Reverse Transcriptase (Invitrogen), according to the manufacturer’s recommendations. SYBR Green RT-qPCR was performed to evaluate the mRNA levels in each sample (Platinum SYBR Green qPCR SuperMix-UDG, Invitrogen) using the Step One Plus Real-time PCR system (Applied Biosystems). After an initial denaturation at 95 °C for 2 min, the thermal cycles were repeated 40 times as follows: 95 °C for 15 s and 60 °C for 30 s. The housekeeping genes glyceraldehyde 3-phosphatase dehydrogenase (*GAPDH*) and ribosomal protein S18 (*RPS18*) were used to normalize the cDNA levels. The sequences of the human primers used for gene amplification are shown in Supplementary Table 1.

### DNA methylation analysis

COBRA^47^ was used to assess the methylation status of the specific CpG sites located in the promoter regions of somatostatin (*SST*) and insulin (*INS*). An in silico analysis using the UCSC Genome Bioinformatics Site (http://genome.ucsc.edu) was performed to identify the CpG sites associated with the proximal promoter for each gene. A proximal CpG was defined as a CpG located within 500 bp (±) of the transcription start site. MethPrimer (http://www.urogene.org/methprimer) was used to design the COBRA primers required to amplify the genomic regions containing the CpG of interest (Supplementary Table 2). Briefly, 1 µg of genomic DNA was subjected to bisulfite modification treatment using the EpiTect Plus kit (QIAGEN). Then, COBRA PCR was performed as follows: after an initial denaturation step at 94 °C for 3 min, the following thermal cycles were repeated 40 times: 94 °C for  10 s, 55 °C for 50 s, and 72 °C for 1 min. Each COBRA PCR was performed in a total volume of 10 µL, which contained 0.5 units of Hot Start Taq polymerase (Takara), 10 pmol of primers, and 1 µL of bisulfite-treated DNA. After PCR amplification, 3 µL of amplified products were digested with three units of restriction enzyme. Finally, the restriction products were separated by 10% PAGE and visualized by ethidium bromide staining. The bands were densitometrically analyzed using the software ImageJ (v1.38×, National Institutes of Health, USA; http://rsb.info.-nih.gov/ij) to quantify the unmethylated (U) and methylated (M) restriction fragments. The methylation levels were calculated for each locus using the formula (M × 100)/(M + U) and were expressed as a methylation percentage.

### In vivo epigenetic reprogramming

To determine the optimal dose of the demethylating drug for in vivo administration, the mice received a daily IP injection of 5-AZA diluted in sterile saline solution at concentrations ranging from 1.5 to 15 mg/kg (*N* = 5 for each concentration) 6 times/week. The animals’ conditions and weights were monitored twice a week. For in vivo epigenetic reprogramming, the PDAC cells were first implanted in athymic nude mice as described above. After the tumors reached a palpable size (≥100 mm^3^), the animals were added to the study and randomly separated into two groups. Mice that did not develop tumors were excluded from the study. Next, the mice received an IP injection of 3 mg/kg 5-AZA (*N* = 8) or saline solution (*N* = 8) 6 times/week for 4 weeks. The tumor size was monitored twice a week, as described above. The animals were euthanized at the study endpoint dictated by the animal care guidelines of our institute. The tumors were immediately removed and snap frozen in liquid nitrogen for storage until RNA and DNA extraction.

### Statistical analysis

The experimental data are presented as the means ± SD, except for the in vivo tumorigenicity assay, in which error bars show the SEM. Student’s *t*-test was performed to estimate the statistical significance of the data. The equality of the variances was tested using an *F*-test, and correction was performed in the case of unequal variances (Welch’s *t*-test). All *p*-values were two-tailed. The correlations between the gene expression (RT-qPCR) and DNA methylation levels (COBRA) were assessed by calculating the Spearman’s rank coefficient. All statistical analyses were performed using the MedCalc software. The experimental data are representative of at least three independent experiments and were considered statistically significant at a *p* < 0.05.

## Electronic supplementary material

Supplementary Information
