# Supplementary material for: Epigenetic reprogramming using 5-azacytidine promotes an anti-cancer response in pancreatic adenocarcinoma cells
Source: Cell Death Dis. 2018 Apr 27;9(5):468. doi: 10.1038/s41419-018-0487-z (PMC5920091; doi:10.1038/s41419-018-0487-z)
Supplement: Supplementary file 1 — Supplementary Information [file 41419_2018_487_MOESM1_ESM.pdf]

SUPPLEMENTARY INFORMATION

Epigenetic reprogramming using 5-azacytidine promotes an anti-cancer response in pancreatic adenocarcinoma cells

Luc Gailhouste[1], Lee Chuen Liew[1,2], Izuho Hatada[3], Hitoshi Nakagama[2,4], and Takahiro Ochiya[1]

[1]Division of Molecular and Cellular Medicine, National Cancer Center Research Institute, Tokyo, Japan; [2]Graduate School of Medicine, The University of Tokyo, Tokyo, Japan; [3]Laboratory of Genome Science, Biosignal Genome Resource Center, Institute for Molecular and Cellular Regulation, Gunma University, Maebashi, Japan; [4]National Cancer Center, Tokyo, Japan.

Corresponding author: Luc Gailhouste or Takahiro Ochiya, Division of Molecular and Cellular Medicine, National Cancer Center Research Institute, 5-1-1 Tsukiji, Chuo-ku, Tokyo 104-0045, Japan. Tel: +81-3-3542-2511. Fax: +81-3-3543-9305. E-mail: lgailhou@ncc.go.jp; tochiya@ncc.go.jp.

Supplementary Table 1. List of the human primers used for real-time quantitative PCR.

Gene	Name	Sense	Antisense
GAPDH	Glyceraldehyde 3-phosphatase dehydrogenase	gagtcaacggatttggtcgt	ttgattttggagggatctcg
RPS18	Ribosomal protein S18	gaggatgaggtggaacgtgt	ggacctggctgtattttcca
DNMT1	DNA methyltransferase 1	gagctaccacgcagacatca	cgaggaagtagaagcggttg
INS	Insulin	ccctgcagaagcgtggcatt	ccatctctctcggtgcagga
GCG	Glucagon	catttactttgtggctggat	cgcttgtcctcgttcatctg
PDX1	Pancreatic and duodenal homeobox 1	cctttcccatggatgaagtc	ttcaacatgacagccagctc
IAPP	Islet amyloid polypeptide	gaaatgcaacactgccacat	attggatcccacgttggtag
SST	Somatostatin	ccaaccagacggagaatgat	ccatagccgggtttgagtta
SSTR1	Somatostatin receptor 1	ggcgaaatgcgtcccag	cggagtagatgaaagagatcagga
SSTR2	Somatostatin receptor 2	gtcctctgcttggtcaaggtg	tggtctcattcagccgggatt
SSTR3	Somatostatin receptor 3	atgcttcatccatcatcggtgtc	ggggatcagaacgccactga
SSTR4	Somatostatin receptor 4	ggaggagcccctggactac	gccgggttctggttgcag
SSTR5	Somatostatin receptor 5	gcctgggtcctgtctctgtg	taccgccctcctgcacgt

The primers were designed using Primer3 v.0.4.0 (http://bioinfo.ut.ee/primer3-0.4.0/primer3) and purchased from Invitrogen. The primers for SSTRs were designed according to the study of Li and coworkers to avoid the homology between the different subtypes (Li et al., 2004).

Supplementary Table 2. Human primers used for COBRA.

Target name	Sense	Antisense
INS	gggggttgaggttgtaatttt	aacatttacccccaactataaacaa
SST	tggagagtggttggttaaattttag	aatcttatcacctcccctatcttctata

All primers were designed using the UCSC Genome Bioinformatics Site (http://genome.ucsc.edu) and MethPrimer (http://www.urogene.org/methprimer, Li and Dahiya, 2002) and were purchased from Invitrogen.

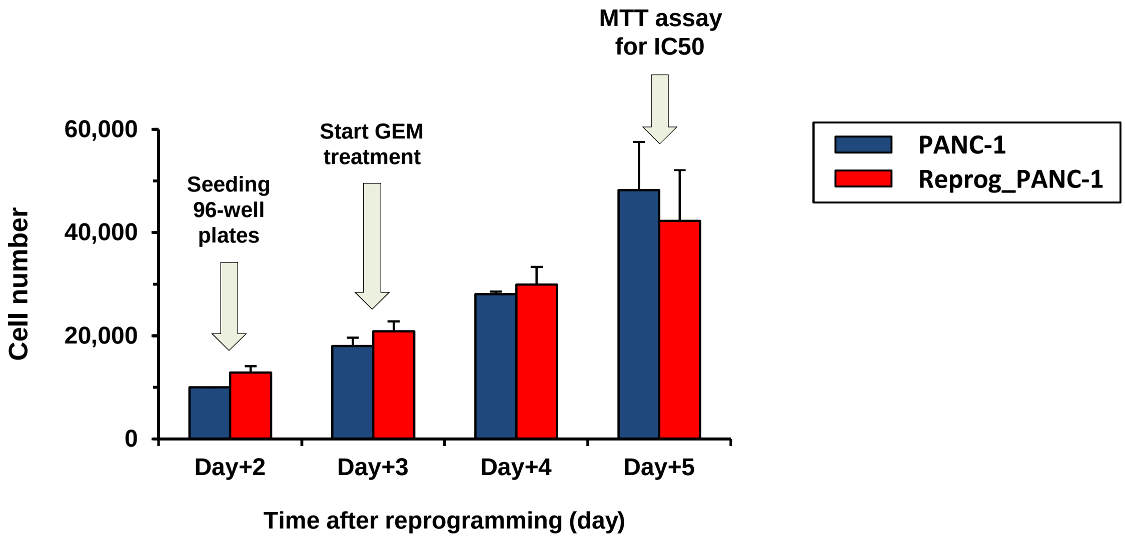

Supplementary Figure 1

Supplementary Figure 1. PANC-1 cell growth after epigenetic reprogramming. PANC-1 cells were pretreated with 3 µM 5-AZA for two weeks with daily replacement. After 48 hours without 5-AZA, the reprogrammed and control cells were seeded in 96-well plates. Cell number was estimated at the indicated times using a cell viability assay (MTT). The data depicted show the mean of three distinct experiments ± standard deviation (SD). The differences between the reprogrammed and control PANC-1 cells were evaluated using the *t*-test, and statistical significance was not observed. The light green arrows depict the experimental layout for the measurement of GEM cytotoxicity on PANC-1 cells after epigenetic reprogramming.

Somatostatin - *SST*

TISSUE | RNA CELL | CANCER

PANCREAS - Expression summary			
Protein expression	n l m h		
RNA expression	HPA:	78.7	TPM
	GTEx:	162.7	RPKM
	FANTOM5:	709.2	Tags Per Million

PANCREAS - Annotated protein expression		
Annotated protein expression	Exocrine glandular cells:	Not detected
	Islets of Langerhans:	High

Antibody staining	Antibody HPA019472	Antibody CAB000075
Exocrine glandular cells	Not detected	Not detected
Islets of Langerhans	High	Medium

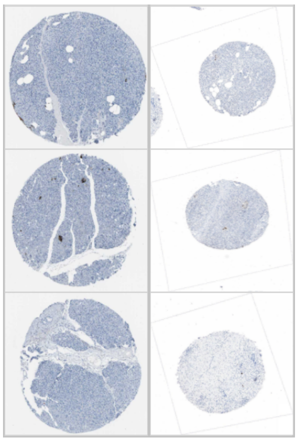

from The Human Protein Atlas
http://www.proteinatlas.org/ENSG00000157005-SST/tissue/pancreas

Positive for SST in 6/6 pancreas (normal tissue)

Somatostatin - *SST*

TISSUE | RNA CELL | CANCER

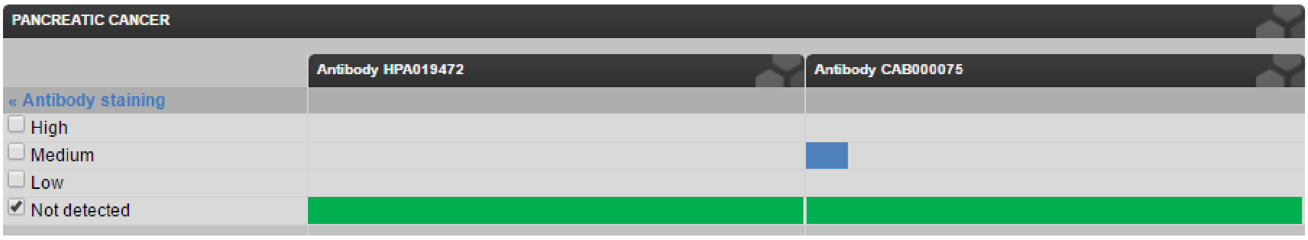

Positive for SST in 0/9 pancreatic adenocarcinoma tissues

Positive for SST in 1/12 pancreatic adenocarcinoma tissues

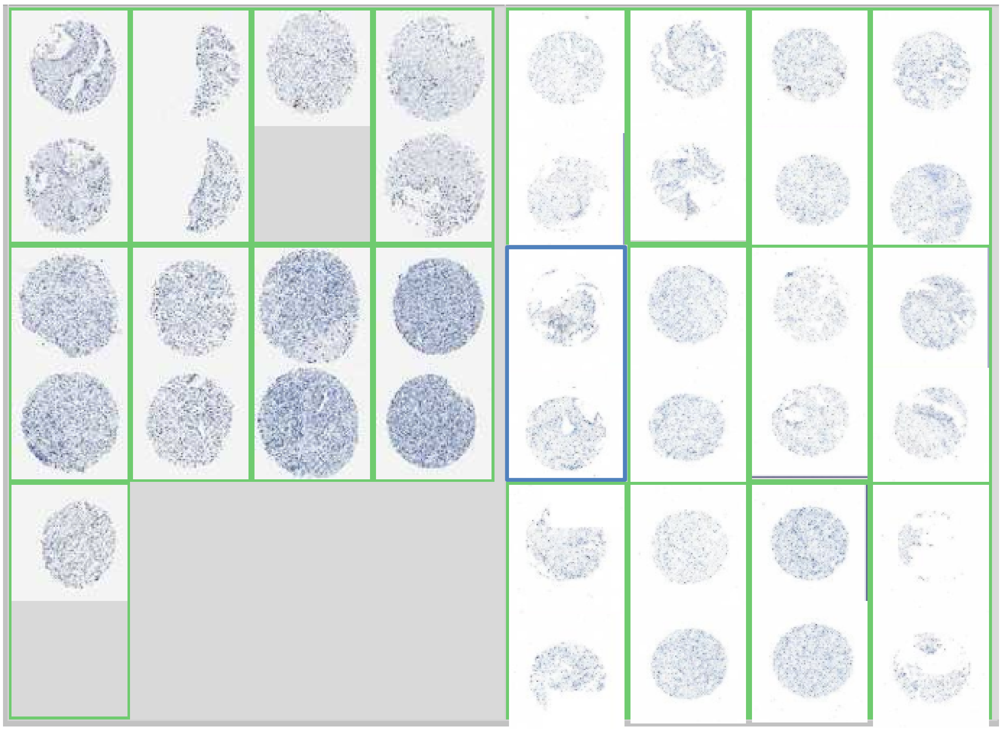

from The Human Protein Atlas
http://www.proteinatlas.org/ENSG00000157005-SST/cancer/tissue/pancreatic+cancer#Quantity

Supplementary Figure 2

Supplementary Figure 2. SST immunostaining in pancreatic adenocarcinoma clinical samples and normal pancreas. The data were obtained from the Human Protein Atlas Program (Uhlén et al., 2015). The figure shows SST antibody-based protein profiling using immunochemistry assays. The 6 control pancreatic tissues were positive for SST using two distinct antibodies (N=3 for each antibody). SST protein was not detected in 9 and 11 pancreatic adenocarcinoma samples using the first (total samples N=9) and second antibody (total samples N=12), respectively. Further information on SST antibodies and patients are available on the Human Protein Atlas website (www.proteinatlas.org).

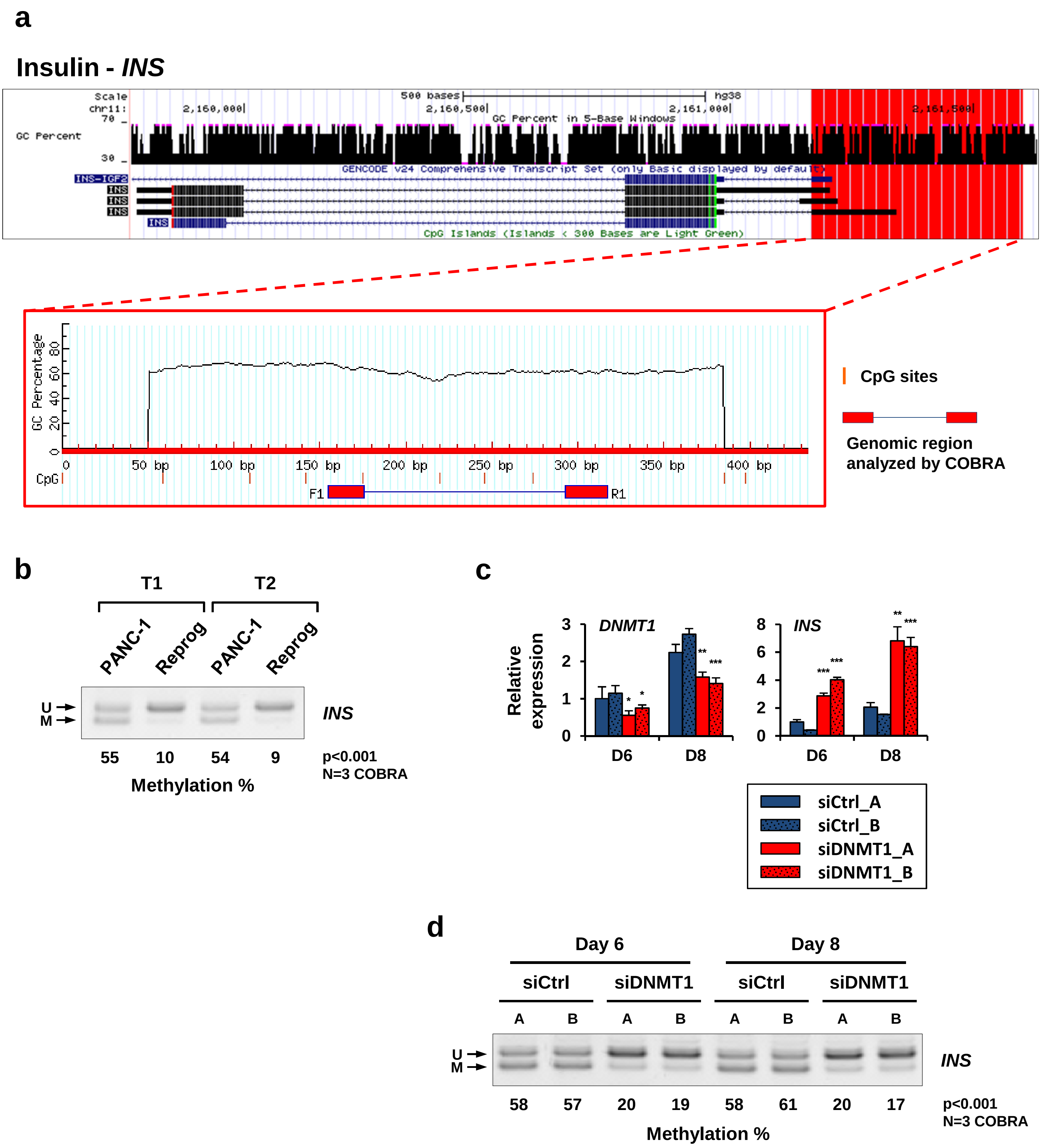

Supplementary Figure 3

Supplementary Figure 3. Expression levels and promoter methylation profiles of *INS* after 5-AZA treatment and DNMT1-knockdown. (a) *In silico* analysis of the human *INS* gene. The figures show the GC percentages, CpG sites, and the COBRA-amplified genomic region. The CpG sites in the *INS* promoter were identified using the UCSC Genome Bioinformatics Site (http://genome.ucsc.edu/). No CpG island was found but a CpG-rich region was identified in *INS* promoter. **(b)** COBRA was performed to evaluate CpG methylation (%) in the promoter of *INS* gene in the control and epigenetically reprogrammed PANC-1 cells. Reprogrammed cells were treated with 5-AZA (3 µM) for 14 and 16 days (T1 and T2, respectively) before genomic DNA extraction. Representative data of three COBRA are shown. **(c)** Relative expression of *DNMT1* and *INS* following *DNMT1* silencing in PANC-1 cells. Two distinct siRNAs were used to target *DNMT1* (siDNMT1_A and B), and 2 scrambled siRNAs were used as negative controls (siCtrl_A and B). The histograms show the mean ± SD of *INS* and *DNMT1* expression levels, measured 6 and 8 days after transfection. **(d)** *INS* promoter methylation after *DNMT1* knockdown, as determined by COBRA. The data are representative of three COBRA. Genomic DNA was extracted from PANC-1 cells 6 and 8 days after transfection. Statistical significance: *$p<0.05$, **$p<0.01$, and ***$p<0.001$ (*t*-test). U, unmethylated; M, methylated.

a

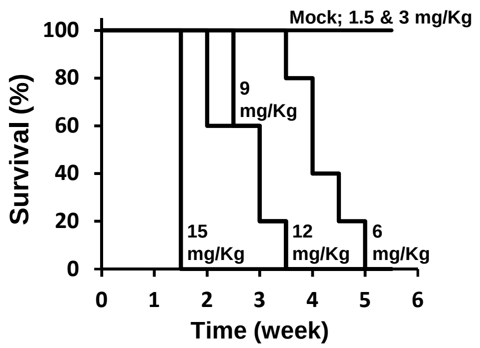

b

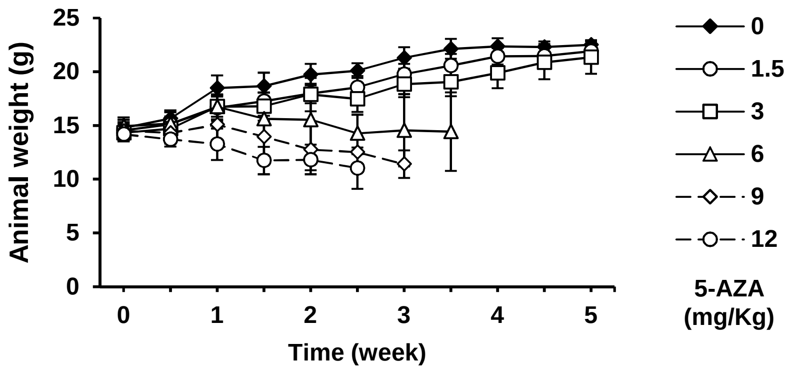

Supplementary Figure 4

Supplementary Figure 4. Survival and weight of the animals treated with 5-AZA. (a) Survival in response to 5-AZA treatment. The mice were injected with 5-AZA at the indicated doses 6 times/week (IP) for 5 weeks. Each dose was tested with 5 mice. **(b)** Animal weight in response to 5-AZA. The weight of each mouse was monitored twice a week. Animal experiments were performed in accordance with the regulations of the National Cancer Center Institutional Animal Care and Use Committee.

Somatostatin receptor 1 – *SSTR1*

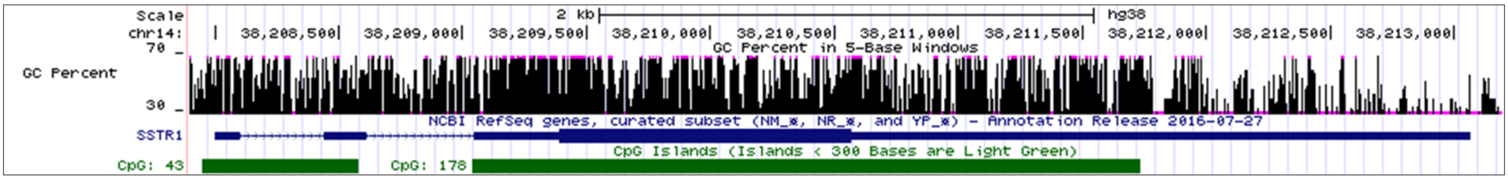

Somatostatin receptor 2 – *SSTR2*

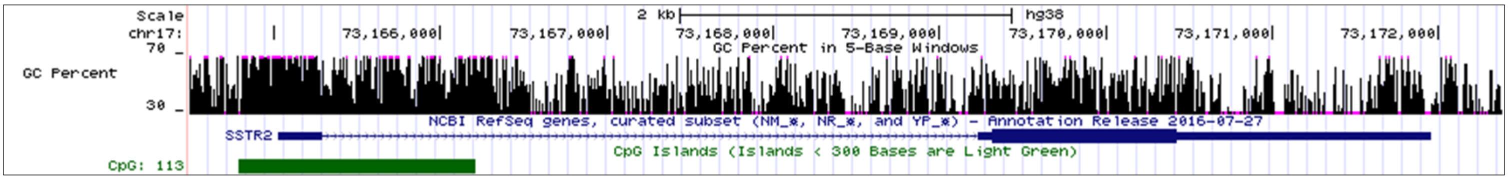

Somatostatin receptor 3 – *SSTR3*

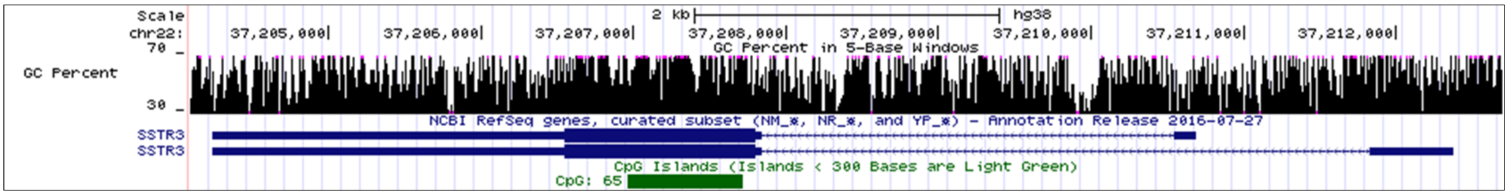

Somatostatin receptor 4 – *SSTR4*

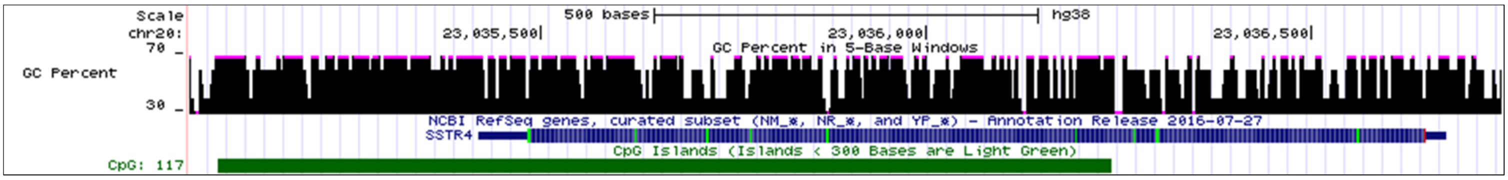

Somatostatin receptor 5 – *SSTR5*

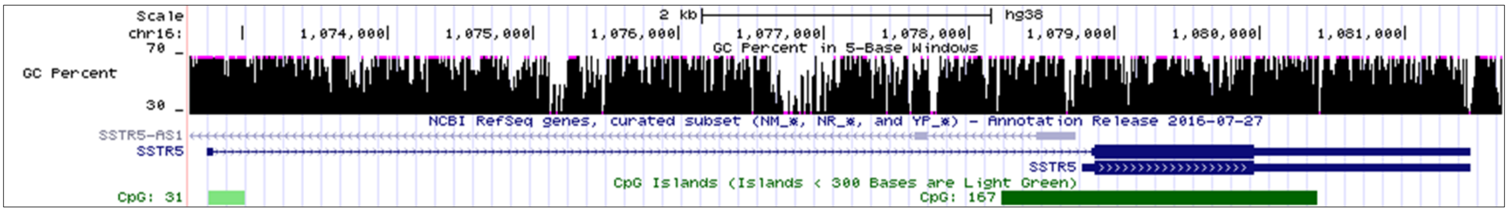

Supplementary Figure 5

Supplementary Figure 5. *In silico* analysis of the five human *SSTR* genes. The figure shows the GC percentages and CpG islands (green horizontal bars) for each gene. The data were extracted from the UCSC Genome Bioinformatics Site (http://genome.ucsc.edu/). CpG islands ranging from 31 to 178 bp were identified in the five genes.

SUPPLEMENTARY REFERENCES

Li, L.C. & Dahiya, R. MethPrimer: designing primers for methylation PCRs. *Bioinformatics* **18**, 1427-1431 (2002).

Li, M. et al. Characterization of somatostatin receptor expression in human pancreatic cancer using real-time RT-PCR. *J. Surg. Res.* **119**, 130-137 (2004).

Uhlen, M. et al. Proteomics. Tissue-based map of the human proteome. *Science* **347**, 1260419 (2015).
